# A retrospective analysis of budget impact models submitted to the National Centre for Pharmacoeconomics in Ireland

**DOI:** 10.1007/s10198-020-01181-0

**Published:** 2020-03-30

**Authors:** Felicity Lamrock, Laura McCullagh, Lesley Tilson, Michael Barry

**Affiliations:** 1 Queen’s University Belfast, Belfast, UK; 2 National Centre for Pharmacoeconomics, St James’s Hospital, Dublin, Ireland; 3 Department of Pharmacology and Therapeutics, Trinity College Dublin, Dublin, Ireland

**Keywords:** Budget impact analysis, Buget impact model, Health Technology Assessment, Reimbursement, New drugs, Ireland, H61 Budgets, E42 Payment Systems, L65 Drugs, O52 Europe, G18 Government Policy and Regulation, C60 General

## Abstract

**Background:**

The National Centre for Pharmacoeconomics (NCPE) is a National HTA Agency in Ireland responsible for assessment of comparative clinical effectiveness, cost-effectiveness and potential budget impact of drugs on behalf of the Health Service Executive. This research aims to assess if the budget impact models submitted to the NCPE have accurate predicted utilisation, assess if the models are consistent in the parameters included, and determine if probabilistic sensitivity analyses would aid the characterization of uncertainty.

**Methods:**

A retrospective analysis of budget impact models that had been submitted (January 2010–December 2017 inclusive) to the NCPE was performed. The input parameters in the budget impact model were recorded. For each drug, annual realised utilisation was compared with what had been predicted by the respective budget impact model. A probabilistic sensitivity analysis was also performed on each model.

**Results:**

A total of 12 models were included; each model pertained to one drug for one indication. Of the 12 models, six underpredicted and six overpredicted the annual realised utilisation. There were a range of different parameters included in each of the budget impact models. A probabilistic sensitivity analysis did not improve the characterization of uncertainty.

**Conclusion:**

This research has demonstrated that budget impact models submitted to a national HTA agency have limited accuracy in predicting realised utilisation, and there is inconsistency among the parameters included. An electronic budget impact template for applicants has been developed, as a more systematic approach, for their submissions to the NCPE.

## Introduction

In Ireland, all new drugs for which reimbursement by the healthcare payer [the Health Service Executive (HSE)] is sought, are considered for Health Technology Assessment (HTA). The National Centre for Pharmacoeconomics (NCPE) [1] is a national HTA agency that is commissioned by the HSE to conduct HTAs. An HTA is a multidisciplinary process that involves a robust, unbiased, systematic, evidence-based assessment of the comparative effectiveness, cost-effectiveness and potential budget impact of new drugs [1]. In Ireland, the Health (Pricing and Supply of Medical Goods) Act 2013 [2] places, on a legal footing, the criteria the HSE must consider when making pricing and reimbursement decisions. Cost effectiveness is named as one such criterion. Cost effectiveness is evaluated within a defined framework for the pricing and reimbursement of new drugs. This framework is described within the HSE-Irish Pharmaceutical Healthcare Association (IPHA) Agreement [3]. It is referenced to within the HSE’s National Service Plan 2020. The main elements of the agreement include detailing of the national pricing framework agreements, supply arrangements, and the principles and processes for reimbursement for new drugs [3].

The applicant pharmaceutical company (herein ‘the applicant’) submits an HTA dossier to the NCPE which includes a cost-effectiveness model and a budget impact model (both fully functional). The NCPE technology review group critically evaluates the applicant submission and provides the HSE (decision maker) with a final appraisal report. This report is comprehensive and details, among other criteria, cost effectiveness by standard decision rules, the expected budget impact, and also provides the decision maker with a reimbursement recommendation. The NCPE evaluation process has been described in more detail elsewhere [4].

Budget impact analysis (BIA) has been defined as a tool to predict the potential financial impact of the adoption of a new technology into a healthcare system with finite resources over a finite number of years [5–6]. In Ireland, a BIA may be used to provide information to inform the HSE on the affordability of a new drug. In the HSE–IPHA agreement, it is stated that in line with statutory obligations, the HSE operates within the resources provided by the Government (Dáil Éireann), each year [3]. The Health (Pricing and Supply of Medical Goods) Act 2013 requires that the decision maker (the HSE) also considers the affordability as part of the reimbursement-decision process [2]. Resources are limited, and it is unclear whether the budget impact models submitted by the applicants tend to overestimate or underestimate the real-world budget impact in Ireland. However, it is known that there are challenges with forecasting budget impact in other countries, in particular, assumptions about uptake can greatly influence the accuracy of results [7]. We believe that the estimation of the real-world budget impact in Ireland (as part of the HTA) should be monitored closely, given that the HSE’s capacity to fund new drugs is limited.

As part of the HTA process, the NCPE assesses the budget impact models submitted by the applicants in accordance with the national HTA guidelines [6] and in conjunction with the NCPE applicant template for HTA submissions [8]. The national HTA guidelines outline how a consistent methodological approach is required to compare drugs between disease areas over time. The guidelines also describe the need for a probabilistic sensitivity analysis to provide decision makers with information regarding the sensitivity of the budget impact model to specific assumptions [6]. The NCPE applicant template for HTA submissions conversely only requests a one-way sensitivity analysis and a scenario analysis [8]. Also, other task force guidelines do not support the use of a probabilistic sensitivity analysis in budget impact analyses [5]. However, a probabilistic sensitivity analysis may prove useful for the decision maker as it has the potential to provide a more realistic assessment of parameter uncertainty than other types of sensitivity analysis [9].

In Ireland, the HSE Primary Care Reimbursement Service (PCRS) prescription database records details of prescription drugs dispensed to patients in the community setting (on the community drugs schemes) [10]. There are several different community drug schemes in Ireland through which drugs are dispensed in the community via community pharmacies. Drugs dispensed through the various schemes attract different rebates and fees. The HSE PCRS community drug scheme prescription database captures all drug utilisation data including brand name, strength, formulation, pack size, and monthly utilisation. Rebates, pharmacy fees and VAT are also recorded. The HSE PCRS community drugs scheme prescription database is described in more detail elsewhere [11].

The objective of this work is to perform a retrospective analysis of budget impact models submitted by applicants to the NCPE, to assess if they are accurate in their predictions of the realised utilisation to the HSE. In addition, this work will assess if the budget impact models are consistent in the parameters included, and if a probabilistic sensitivity analysis could better characterise the uncertainty surrounding the predicted impact on the budget in Ireland to support the decision makers on their reimbursement decisions.

## Methods

### HTA selection

All HTAs commissioned by the HSE and submitted to the NCPE between January 2010 and December 2016 inclusive were considered for inclusion. HTAs were excluded if the respective drug was not reimbursed, or had not been reimbursed for a full year. The HSE PCRS community drug scheme prescription database does not capture drugs with a hepatitis C indication or hospital only drugs, and thus these drugs were excluded from this analysis. Similarly, the HSE PCRS community drug scheme prescription database data do not provide data on the indication for which a drug was supplied. We note that the NCPE obtains separate budget impact models for each indication for each drug. Thus, drugs which were reimbursed for multiple indications were excluded. The analysis was performed in December 2017. January 2010 was chosen as the study period start date. In Ireland, 2010 was the first full year that all drugs, for which reimbursement by the HSE was sought, were considered for HTA.

### Model recreation

Budget impact models submitted to the NCPE consist of a gross drug budget impact, a net drug budget impact, and an overall net budget impact. For the HTAs within the NCPE, a gross drug budget impact is defined as containing only the new drug acquisition costs, which is based on assumptions regarding the eligible patient population, market share, and drug cost for 5 years. A net drug budget impact describes the potential costs and cost offsets from the displacement of other drugs. An overall net budget impact takes into account other costs such as costs of administration or concomitant medication [8].

To be able to compare realised utilisation with the predicted utilisation from applicant submissions, each individual budget impact model was recreated, and the gross drug budget impact only was used. The models were recreated to validate the applicant’s submission as well as enabling the macros (which were created for a probabilistic sensitivity analysis) to be easily adapted for each model. Each individual gross drug budget impact model was recreated and programmed in Microsoft Excel^®^ 2010.

### Resource utilisation

A retrospective analysis (January 2010–December 2017 inclusive) of the HSE PCRS community drug scheme prescription database (data anonymised) was performed. These data represent realised utilisation. For each drug, the quantity of the drug paid for by the HSE each month within the dates of the analysis was calculated. The information analysed from the data was the date, the drug reimbursed and the drug expenditure (starting from the month of reimbursement) over a 5-year period or part there of (if the drug had been reimbursed for less than 5 years).

For the purposes of this analyses, the drug acquisition cost was defined as the applicant’s list price of the drug to the pharmacy. This definition was used in both the analysis of the realised utilisation and the predicted utilisation of applicants’ budget impact models for a direct comparison. Rebates, pharmacist fees, and VAT were not included in the analysis due to confidentially issues, and therefore, only the publicly available list price (at which the drug was reimbursed from HSE) was entered into the recreated budget impact models. Due to confidentiality, the actual values cannot be used, and so the total annual predicted budget impact for each drug each year is presented as a percentage of the total annual realised utilisation.

### Assessment of parameters included

The components of the budget impact models consisted of several types of parameters. The total number of input parameters in each of the budget impact models was recorded and the parameter inputs were categorised as pertaining to ‘Population’, ‘Market Share’, ‘Dosage’ and ‘Acquisition Costs’. The parameters that were used to calculate the applicant’s eligible population were categorised under ‘Population’ [8]. The ‘Market Share’ parameters consisted of percentages (in each of the 5 years) of how many patients from the eligible patient population who would be expected to receive the drug under consideration and could include parameters involving switching from a comparator drug to the new drug. The ‘Dosage’ parameters relate to the daily, weekly, monthly or annual dosage of the new drug to calculate how many packs or vials of the new drug are required by one patient per year. The ‘Acquisition Costs’ parameters relate to the cost of the new drug. The models were also investigated to assess what specific modelling parameters from the four parameter input categories were included that are often not explicitly included in budget impact models submitted to the NCPE: Incidence, Prevalence, Population growth, Adherence, Discontinuation, and Duration of treatment.

### Probabilistic sensitivity analysis

A probability distribution outlines the relative likelihood that a range of values is the true value of a parameter. A probabilistic sensitivity analysis assigns an informed probability distribution to the key parameters in a budget impact model to capture uncertainty in the results [6]. Samples are drawn at random from the corresponding distributions through a large number of simulations. The Monte Carlo simulation method is used here with an arbitrary 1000 simulations.

A macro programmed in Excel Visual Basic for applications was written and inserted into each of the recreated budget impact models to perform a probabilistic sensitivity analysis. All analyses were conducted in Microsoft Excel^®^ 2010.

A range of plus or minus 20 percent using a uniform distribution (which assigns equal probability to all parameters in the range) was used due to the high level of uncertainty of the true value being the top of a bell-shaped probability distribution. The respective cost-effectiveness models had previously been validated by the NCPE review group in terms of their parameter assumptions and characteristics of uncertainty. All of the parameters apart from the agreed list price of the drug were varied in the probabilistic sensitivity analysis.

## Results

### HTAs included

A total of 113 HTAs for drugs (that had been commissioned by the HSE and evaluated by the NCPE) were identified between 2010 and December 2017 inclusive. Twenty one HTAs were excluded as (contrary to NCPE requirements) there was no budget impact model available, 37 HTAs were excluded as the drugs had multiple indications and so no accurate estimate of the relevant budget impact would be measurable, 28 HTAs were excluded as the drug was not reimbursed or was not reimbursed for a least one full year, 10 HTAs were excluded due to being hospital only drugs, and 5 HTAs were excluded due to having hepatitis C indications.

**Fig. 1:**
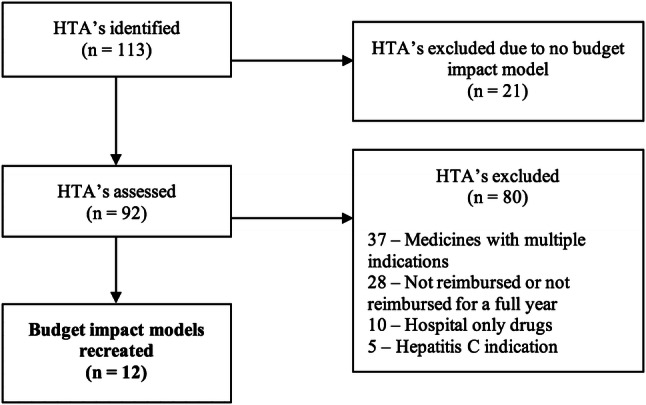
Diagram of budget impact models included to be recreated

A total of 12 HTAs were included in the analysis (Fig. 1). As the HTA submissions from the applicants is confidential, each drug is identified by letters A–L.

### Resource utilisation

Table 1 outlines the 12 HTAs included by letters A–L and the annual percentage over- or underprediction of the total predicted utilisation (at the list price) that the recreated budget impact model predicted compared to the realised utilisation. Only one drug was reimbursed for all 5 years of comparison (drug F), whereas one drug was reimbursed for 1 year (drug L). The recreated models for drugs A, B, H, J, L overpredicted in all of the years compared. The recreated models for drugs D, E, F, I, K underpredicted in all of the years compared. The recreated models for drugs C and G either over- or underpredicted annually. When all 12 models are considered, the predictions ranged from an overprediction of 94% in 1 year for one of the drugs to an underprediction of 1017% in another year for a different drug.

**Table 1:** Drugs included in the analysis and budget impact model prediction results

Drug	Year 1	Year 2	Year 3	Year 4	Year 5
A	−36%	−57%	N/A	N/A	N/A
B	−49%	−78%	−94%	N/A	N/A
C	420%	22%	−21%	N/A	N/A
D	180%	409%	N/A	N/A	N/A
E	490%	537%	477%	N/A	N/A
F	1017%	585%	334%	219%	165%
G	6%	−25%	−56%	−61%	N/A
H	−49%	−66%	−87%	N/A	N/A
I	72%	79%	228%	N/A	N/A
J	−76%	−72%	N/A	N/A	N/A
K	95%	59%	N/A	N/A	N/A
L	−12%	N/A	N/A	N/A	N/A

The annual percentage over or underprediction of the predicted utilisation (at the list price) that the budget impact model by the applicant made compared to the realised utilisation as a percentage. A positive value indicated a model underprediction, a negative value indicates a model overprediction

*N/A*, not available

^a^Not all drugs have been reimbursed for 5 years as of December 2017 when the analysis was performed, so N/A applies in several instances

### Assessment of parameters included

Table 2 describes the parameters classified in each of the budget impact models. The number of ‘Population’ parameters in the individual models ranged from a total of 3–91; whereas, the number of ‘Acquisition Cost’ parameters was one apart from two cases. In each model, the number of ‘Market Share’ parameters ranged from 5 to 30, with five being the most common (one for each of the 5 years of the budget impact model). The number of ‘Dosing’ parameters in each model ranged from 1 to 4. Table 3 describes the modelling included in each of the budget impact models. Five of the budget impact models modelled incidence, all included prevalence, eight modelled population growth, one modelled adherence, two modelled discontinuation and three modelled duration of treatment.

**Table 2:** Parameters classified in each of the budget impact models

Drug	Population	Market share	Dosage	Acquisition costs
A	4	5	4	1
B	7	5	1	1
C	15	30	1	2
D	14	10	1	1
E	3	12	2	1
F	91	5	2	3
G	21	15	1	1
H	5	5	1	1
I	10	5	1	1
J	8	5	2	1
K	5	5	1	1
L	4	5	1	1

**Table 3:** Specific modelling parameters included in each of the budget impact models

Drug	Incidence	Prevalence	Population growth	Adherence	Discontinuation	Duration of treatment
A	No	Yes	Yes	No	No	No
B	No	Yes	Yes	No	No	No
C	No	Yes	Yes	Yes	No	No
D	Yes	Yes	No	No	Yes	No
E	Yes	Yes	No	No	No	Yes
F	No	Yes	Yes	No	No	No
G	No	Yes	Yes	No	No	No
H	Yes	Yes	No	No	No	Yes
I	Yes	Yes	Yes	No	Yes	No
J	Yes	Yes	Yes	No	No	Yes
K	No	Yes	Yes	No	No	No
L	Yes	Yes	No	No	No	No

### Probabilistic sensitivity analysis

The probabilistic sensitivity analysis (PSA) was performed on each of the models. For each full year, the utilisation data (from the HSE PCRS community drug scheme prescription database) was compared to the upper and lower bounds of the probabilistic sensitivity analysis. If the realised utilisation was situated between the minimum and maximum probabilistic sensitivity analysis value, the year and drug was given a status “inside PSA bounds”. The status “outside PSA bounds” was assigned where the probabilistic sensitivity analysis failed to capture within its bounds the realised utilisation. Table 4 describes whether the probabilistic sensitivity analysis captured the realised utilisation. N/A was applied in the instances where no yearly data existed due to the drug being reimbursed for less than 5 years. Only three probabilistic sensitivity analyses consistently captured the realised utilisation in the bounds of the probabilistic sensitivity analysis. Other distributions were investigated and made little difference to the results (not reported).

**Table 4:** Probabilistic sensitivity analysis results

Drug	Year 1	Year 2	Year 3	Year 4	Year 5
A	Inside PSA bounds	Inside PSA bounds	N/A^a^	N/A	N/A
B	Inside PSA bounds	Outside PSA bounds	Outside PSA bounds	N/A	N/A
C	Outside PSA bounds	Inside PSA bounds	Inside PSA bounds	N/A	N/A
D	Outside PSA bounds	Outside PSA bounds	N/A	N/A	N/A
E	Outside PSA bounds	Outside PSA bounds	Outside PSA bounds	N/A	N/A
F	Outside PSA bounds	Outside PSA bounds	Outside PSA bounds	Outside PSA bounds	Outside PSA bounds
G	Inside PSA bounds	Inside PSA bounds	Inside PSA bounds	Outside PSA bounds	N/A
H	Inside PSA bounds	Outside PSA bounds	Outside PSA bounds	Outside PSA bounds	N/A
I	Inside PSA bounds	Inside PSA bounds	Inside PSA bounds	N/A	N/A
J	Outside PSA bounds	Outside PSA bounds	N/A	N/A	N/A
K	Outside PSA bounds	Inside PSA bounds	N/A	N/A	N/A
L	Inside PSA bounds	N/A	N/A	N/A	N/A

Inside PSA bounds—the realised utilisation was situated between the minimum and maximum probabilistic sensitivity analysis value; outside PSA bounds—the realised utilisation was not situated between the minimum and maximum probabilistic sensitivity analysis value

*PSA*, probabilistic sensitivity analysis

^a^Not all drugs have been reimbursed for 5 years as of December 2017 when the analysis was performed, so N/A applies in several instances

## Discussion

In this research, a retrospective analysis of budget impact models was performed by recreating models submitted to the NCPE and comparing the predicted utilisation to the realised utilisation. It was shown that the estimation of the gross drug budget impact of a new drug for which reimbursement is sought in Ireland is difficult. There is much inconsistency in the parameters included in each of the models which may be a potential reason for such inaccurate predictions. Building a regression model to assess the parameters that influence the outcome of the realised utilisation was not possible given the limited number of models assessed; however, the NCPE are evaluating if this would be possible in the future.

Probabilistic sensitivity analyses were performed in an attempt to establish if they could aid the characterization of uncertainty surrounding the predicted impact on the budget in Ireland. The probabilistic sensitivity analysis results showed that the models varied in their ability to predict the realised utilisation within a certain bound. Even when the realised utilisation was situated between the minimum and maximum probabilistic sensitivity analysis value, it is unclear how helpful this would be to the decision maker. A uniform distribution was chosen for the parameters; however, other distributions failed to make little difference to the results. As the ‘Population’ parameters in the models had the widest range of numbers included, further research to determine it is the ‘Population’ parameters that are most often causal (in terms of lack of accurate predictions) might be useful. The NCPE are also evaluating if further earlier research to determine the probability of the realised utilisation being over- or underpredicted would be useful.

According to the Health (Pricing and Supply of Medical Goods) Act 2013, the HSE are required to re-assess a drug within 3–5 years post-reimbursement [2]. The HSE may also at any time re-assess and/or remove the reimbursed status of a drug. Also, in Ireland, the Medicines Management Programme manages a number of post-reimbursement initiatives (with the aim of promoting safe, effective, and cost-effective prescribing). Our outputs (as illustrated in Tables 1, 4) which indicate the lack of accuracy of applicant budget impact models and also highlight the opportunity for early detection of utilisation trends will inform the decision-making of these stakeholders.

The National Guidelines for conducting budget impact analysis in Ireland state that “the purpose of these [budget impact] guidelines is to standardise the method of performing and presenting BIA conducted in Ireland, so that decision makers can be provided with assessments that are reliable, consistent and relevant to their needs.” [12]. This research has demonstrated that the estimation of the budget impact of a new drug for which reimbursement is sought is difficult. The results of this research have led to the creation of an applicant template for the HTA budget impact component of the NCPE assessment. The aim of the template is to provide a more systematic and consistent approach to the budget impact models. The template could have important implications for the HSE if there are more accurate and consistent budget impact predictions. The NCPE anticipate efficiencies for applicants as well as the NCPE through the use of a standardised approach. Explicitly including/excluding certain parameters in the budget impact models would be beneficial for future research which could assess if the template provides a better approach for the budget impact analysis. Additional further research lies in exploring the practical considerations of including a probabilistic sensitivity analysis in the budget impact model template. This template is publicly available at https://www.ncpe.ie/submission-process/submission-templates/budget-impact-model-template/.

The HSE annual budget for new drugs is limited. An underprediction of the budget would have far greater consequences on the limited resources that the HSE have to spend than an overprediction. The HSE state in their National Service Plan 2020 that there is no specific funding in 2020 identified for new drugs. It is, therefore, necessary for the HSE to consider carefully the funding of each recommended drug in the context of available resources and monitor the areas closely, warranting further research into the predictive ability of the budget impact analysis.

One limitation of this analysis was that the gross drug budget impacts were compared, not the net drug budget impacts, which have the potential for further differences in the real-world drug expenditure when rebates, fees, and the list price of comparator drugs are taken into consideration. In addition, the number of drugs included in the analysis was limited.

## Conclusion

This research has demonstrated that there is inconsistency among the parameters included in the budget impact models submitted to the NCPE, a national HTA agency, and models have limited utilisation prediction accuracy compared to the realised utilisation. This information will be useful for our decision makers. An electronic budget impact template for applicants has been developed to attempt to provide a more systematic approach to the models. This may limit uncertainties in predictions of expenditure. Further research may lie in exploring the usefulness and practical considerations of applicants to include a probabilistic sensitivity analysis.
